# Production, secretion and purification of a correctly folded staphylococcal antigen in *Lactococcus lactis*

**DOI:** 10.1186/s12934-015-0271-z

**Published:** 2015-07-16

**Authors:** Frédéric Samazan, Bachra Rokbi, Delphine Seguin, Fabienne Telles, Valérie Gautier, Gilbert Richarme, Didier Chevret, Paloma Fernández Varela, Christophe Velours, Isabelle Poquet

**Affiliations:** INRA, UMR1319 Micalis (Microbiologie de l’Alimentation au service de la Santé), Domaine de Vilvert, 78352 Jouy-en-Josas Cedex, France; Institut Curie/CNRS, UMR3244, 25 rue d’Ulm, 75248 Paris Cedex 05, France; Sanofi Pasteur, Campus Mérieux, 1541 avenue Marcel Mérieux, 69280 Marcy L’Etoile, France; Stress molecules, Institut Jacques Monod, Université Paris 7, 15 rue Hélène Brion, 75013 Paris, France; CNRS, Avenue de la Terrasse, Bât. 34, 91190 Gif-Sur-Yvette, France; LPBA, Institut Pasteur, Bât. Calmette, 75015 Paris, France

**Keywords:** *Lactococcus lactis*, Cell factory, Secretion, *Staphylococcus aureus* antigen, HtrA family, Soluble recombinant protein, Chaperone

## Abstract

**Background:**

*Lactococcus lactis*, a lactic acid bacterium traditionally used to ferment milk and manufacture cheeses, is also, in the biotechnology field, an interesting host to produce proteins of medical interest, as it is “Generally Recognized As Safe”. Furthermore, as *L. lactis* naturally secretes only one major endogenous protein (Usp45), the secretion of heterologous proteins in this species facilitates their purification from a protein-poor culture medium. Here, we developed and optimized protein production and secretion in *L. lactis* to obtain proteins of high quality, both correctly folded and pure to a high extent. As proteins to be produced, we chose the two transmembrane members of the HtrA protease family in *Staphylococcus aureus*, an important extra-cellular pathogen, as these putative surface-exposed antigens could constitute good targets for vaccine development.

**Results:**

A recombinant ORF encoding a C-terminal, soluble, proteolytically inactive and tagged form of each staphylococcal HtrA protein was cloned into a lactococcal expression-secretion vector. After growth and induction of recombinant gene expression, *L. lactis* was able to produce and secrete each recombinant rHtrA protein as a stable form that accumulated in the culture medium in similar amounts as the naturally secreted endogenous protein, Usp45.* L. lactis* growth in fermenters, in particular in a rich optimized medium, led to higher yields for each rHtrA protein. Protein purification from the lactococcal culture medium was easily achieved in one step and allowed recovery of highly pure and stable proteins whose identity was confirmed by mass spectrometry. Although rHtrA proteins were monomeric, they displayed the same secondary structure content, thermal stability and chaperone activity as many other HtrA family members, indicating that they were correctly folded. rHtrA protein immunogenicity was established in mice. The raised polyclonal antibodies allowed studying the expression and subcellular localization of wild type proteins in *S. aureus*: although both proteins were expressed, only HtrA_1_ was found to be, as predicted, exposed at the staphylococcal cell surface suggesting that it could be a better candidate for vaccine development.

**Conclusions:**

In this study, an efficient process was developed to produce and secrete putative staphylococcal surface antigens in *L. lactis* and to purify them to homogeneity in one step from the culture supernatant. This allowed recovering fully folded, stable and pure proteins which constitute promising vaccine candidates to be tested for protection against staphylococcal infection. *L. lactis* thus proved to be an efficient and competitive cell factory to produce proteins of high quality for medical applications.

**Electronic supplementary material:**

The online version of this article (doi:10.1186/s12934-015-0271-z) contains supplementary material, which is available to authorized users.

## Background

*Lactococcus lactis*, a Gram-positive lactic acid bacterium and a classical starter for the manufacture of cheeses, can be used as a cell factory to produce proteins of interest [1–6]. As a long known innocuous, Generally Recognised As Safe (“GRAS”) food-grade species [7], *L. lactis* is an interesting host to produce proteins of medical interest [2, 4, 5]. Compared to *Escherichia coli*, the advantage of *L. lactis* is that it does not produce endotoxin (lipopolysaccharide) [2, 4, 5] which has to be removed from protein preparations before medical use [8]. In contrast to *Bacillus subtilis*, *L. lactis* secretes only one major endogenous protein, Usp45, and no proteases [9]: a strategy combining production and secretion of heterologous proteins in *L. lactis* is thus interesting as it facilitates protein purification from the culture medium [4]. As secreted heterologous proteins can be degraded by the lactococcal surface protease HtrA, protein yield can be improved by the use of a mutant strain devoid of this surface proteolytic activity [10].

Several tools have been developed for protein production in *L.* *lactis*, in particular expression systems and vectors [11–13], secretion signals [4, 14, 15] and expression-secretion vectors [4, 13, 16, 17]. Proteins of medical interest have been successfully produced and secreted by *L. lactis*, in general to be delivered to a host [1, 2], and in a few cases to be purified [16, 18]. In our laboratory, a tightly regulated expression system (ZitR-regulated P_*zit*_ promoter) [11, 19], an efficient export signal (SP_Exp4_ signal peptide) [4, 20], expression-secretion vectors [4, 21] and mutant host strains devoid of surface proteolytic activity [10, 22, 23] have been developed for *L. lactis* and used for protein production and secretion [4, 21, 22, 24]. Furthermore, an enzyme of biotechnological interest, the staphylococcal nuclease, which is naturally secreted, could be produced, secreted and purified in *L. lactis* [21]. Here, we developed, in *L. lactis*, the secretion of high quality proteins for medical applications. We chose, as model proteins, putative surface-exposed antigens and virulence factors: the two HtrA family members of *Staphylococcus aureus*, an important extra-cellular pathogen species.

The HtrA family is composed of highly conserved, extra-cytoplasmic serine proteases [25, 26]. In prokaryotes, they are located in the cell envelope: in either the periplasm or the cytoplasmic membrane in Gram-negative bacteria, and in the cytoplasmic membrane in Gram-positive bacteria. Although some HtrA proteases, like *E. coli* DegS, have a regulatory function, most of them are involved in the protein quality control in the bacterial cell envelope [25–27]. They are often essential for survival to various stress conditions, notably heat [28, 29] and/or oxidative stress [30], because they alleviate protein unfolding and misfolding [25, 27]. They can act both as proteases to degrade proteins and as chaperones to assist them in folding, like *E. coli* DegP/HtrA, the family model [25, 27].

In many pathogens, HtrA proteins are involved in virulence [31]. Several models have been proposed to account for this role. First, HtrA proteases could, under the stress conditions prevailing in the host during infection, degrade unfolded and misfolded proteins and thus indirectly improve cellular fitness and survival [30]. Second, HtrA proteases could play a direct role by processing endogenous, folded, wild type (WT) proteins, as first demonstrated in *L. lactis*, a food-grade species [10, 32] (and unpublished data), and subsequently confirmed in a pathogenic species, *Bacillus anthracis*, even though in that case the HtrA target is not a virulence factor [33]. Third, in some Gram-negative pathogens, HtrA proteases could target host proteins like E-cadherin [34–36] or the interleukin IL8 [37], even if it remains unclear how intra-cellular HtrA proteins are able to reach their extra-cellular host targets [37, 38]. Finally, HtrA proteins could also contribute to virulence as chaperones, either by enhancing in vivo growth and survival, [39], or by improving the folding of a virulence factor [40], or even by contributing to bacteria–host interaction [41].

In pathogens, HtrA proteins are also often important antigens. In several species, they were found to be immunogenic in vivo, in either infected [42–44] or convalescent hosts (animals or human patients) [43–49], even though in *Chlamydia trachomatis* the meaning of these results with respect to disease remains controversial [50, 51]. Moreover, purified HtrA proteins from some Gram-negative pathogens were shown to be protective against infection [45, 49, 52–54] even though this was not always the case [55, 56]. Surprisingly, to our knowledge, no such protection studies have been performed using HtrA proteins of Gram-positive extra-cellular pathogens, despite the fact that, as cell surface exposed proteins, they could be recognized by circulating antibodies at an early infection step and might thus constitute good targets for vaccine development.

In *S. aureus*, an extra-cellular pathogenic species, we previously identified and studied two putative transmembrane members of the HtrA family: HtrA_1_ and HtrA_2_ [23, 57]. WT HtrA_1_ protein from strain RN6390 was found to display extra-cellular proteolytic activity when over-produced in *L. lactis* [23]. Furthermore, both HtrA proteins were, together, implicated in the virulence and extra-cellular proteome composition of strain RN6390, and each of them was involved in the stress resistance of strain COL [57]. In an independent study, among a peptide library from strain COL, a few HtrA_1_ peptides were found to be antigenic and to elicit an immunological response in vivo, in infected patients [48]. As predicted surface-exposed proteins, staphylococcal HtrA proteins from strain COL, and in particular the HtrA_1_ antigen, constitute interesting vaccine candidates [58], and they were thus chosen to be produced in *L. lactis* and purified. Recombinant ORFs encoding soluble, proteolytically inactive and tagged HtrA forms were cloned into a lactococcal expression and secretion vector. Recombinant rHtrA protein yield was evaluated after lactococcal growth in rich medium in flasks or in fermenters after medium optimization. After lactococcal growth in the optimized rich medium in fermenters and induction of gene expression, secreted rHtrA proteins were purified in one step from the culture medium. After confirming their identity my mass spectrometry, rHtrA proteins were analysed in vitro for their secondary structures, stability, oligomeric state and chaperone activity, in order to analyze their folding. Finally, their immunogenicity was tested in mice, and the generated antibodies allowed studying the expression and cell surface exposure of WT HtrA proteins in *S. aureus*.

## Methods

### Bacterial strains and growth conditions

Bacterial strains and plasmids used in this study are described in Table 1. *E. coli* strain JM109 (Promega) and its derivatives carrying plasmids were grown at 37°C, with shaking, in LB medium supplemented with 100 µg/mL ampicillin when necessary for plasmid selection. *L. lactis* strain MG1363 (WT) and its recombinant derivatives carrying plasmids were grown at 30°C in rich M17 medium supplemented with glucose in batch and in a slightly different medium (supplemented by more concentrated glucose and more buffered) in fermenters (see below ‘Recombinant protein production in *L. lactis*’). Chloramphenicol at 10 µg/mL was added when necessary for plasmid selection. *S. aureus* strain Lowenstein (ATCC 49521) and *htrA* mutants of strain COL [57] were grown at 37°C, with shaking, in two media: (1) SATA-2 medium (a Sanofi Pasteur proprietary medium [59]: wheat peptone 93 g/L, d-glucose 0.25 g/L; NaCl 41 g/L, MgCl_2_ 15 g/L), and (2) TSB medium (Difco, Sparks, MD, USA), supplemented or not with 2.2′ Dipyridyl at 1 mM (Sigma-Aldrich).

**Table 1:** Bacterial strains and plasmids used in this study

(A) Bacterial strains		
Names	Genotype, characteristics	Reference
*Escherichia coli*		
JM109	*end*A1, *rec*A1, *gyr*A96, *thi*, *hsd*R17 (r_k_^−^, m_k_^+^), *rel*A1, *sup*E44, Δ(*lac*-*pro*AB), [F´ *tra*D36, *pro*AB, *laq*I^q^ZΔM15]	Promega
*Lactococcus lactis*		
MG1363	plasmid free derivative of NCDO712	Laboratory collection
*S. aureus*		
Lowenstein (ATCC 49521)	clinical isolate, capsular polysaccharide CP5-positive	Laboratory collection
COL	clinical isolate, methicillin resistant	Laboratory collection
*htrA*_*1*_	COL *htrA*_*1*_ :: *cat*, Cm^R^	[57]
*htrA*_*2*_	COL *htrA*_*2*_:: *spc*, Spc^R^	[57]

(B) Plasmids		
Names	Characteristics	Reference
pGEM^T^	T-tailed PCR product Cloning vector, Amp^R^	Promega
pVE8124	pGEM^T^ derivative where recombinant *htrA*_*1r*_ ORF is cloned *htrA*_*1r*_ encodes HtrA_1_-ΔTM-Ser_255_Ala-His_6_ protein	This work
pVE8125	pGEM^T^ derivative where recombinant *htrA*_*2r*_ ORF is cloned *htrA*_*2r*_ encodes SP_Exp4_-HtrA_2_-ΔTM-Ser_619_Ala-His_6_ protein	This work
pLB145	pWV01; Cm^R^; expression-secretion vector where *exp4*_*SP*_-*nuc* is cloned under the control of a lactococcal expression system, P_*zit*_ *zitR* *exp4*_*SP*_-*nuc* encodes a hybrid protein between a lactococcal signal peptide (SP_Exp4_) and the mature secreted form of the staphylococcal nuclease (NucB)	[4]
pVE8126	pLB145 derivative where *htrA*_*1r*_ ORF is cloned in place of *nuc* ORF to be fused in frame to *exp4*_*SP*_ *exp4*_*SP*_-*htrA*_*1r*_ encodes a hybrid precursor leading to secreted rHtrA_1_ protein	This work
pVE8127	pLB145 derivative where *htrA*_*2r*_ ORF is cloned in place of *exp4*_*SP*_ *-nuc* ORF *htrA*_*2r*_ encodes a hybrid precursor leading to secreted rHtrA_2_ protein	This work

*Amp* ^*R*^ ampicillin resistant, *Cm* ^*R*^ chloramphenicol resistant, *Spc* ^*R*^ spectinomycin resistant.

### PCR and cloning

High-fidelity PCRs using chromosomal DNA from *S. aureus* strain COL as a template were performed to obtain recombinant ORFs encoding N-terminally truncated, inactive and tagged forms of staphylococcal HtrA proteins. The recombinant *htrA*_*1r*_ ORF (encoding HtrA_1_-ΔTM-Ser_255_Ala-His_6_) was obtained as follows. (1) The 5′ and the 3′ regions of WT *htrA*_*1*_ gene were amplified by a high-fidelity *Taq* polymerase (FINNZYMME) using respectively the following primer couples (see Additional file 1: Table S1 for primer sequences): 1NFΔTM2 (with a *Nsi*I site at its 5′ end) and 1IRS > A (bearing a point mutation for Ser_255_Ala substitution), or 1IFS > A (bearing a point mutation for Ser_255_Ala substitution) and 1CRHIS (bearing a sequence encoding His_6_ with a limited risk of ribosomal slippage, and bearing an *Eco*RI site at its 3′ end). (2) Using a mix of both the resulting PCR fragments as a template, 1NFΔTM2 and 1CRHIS as primers and a high-fidelity A-tailing *Taq* polymerase (DNA Expand ROCHE), an overlap PCR fragment, *htrA*_*1r*_, was obtained.

The recombinant *htrA*_*2r*_ ORF (encoding SP_Exp4_-HtrA_2_-ΔTM-Ser_619_Ala-His_6_) was obtained as follows. (1) The 5′ and the 3′ regions of WT *htrA*_*2*_ gene were amplified by a high-fidelity *Taq* polymerase (FINNZYMME) using respectively the following primer couples: either 2NFΔTM2 and 2IRS > A (bearing a point mutation for Ser_619_Ala substitution), or 2IFS > A (bearing a point mutation for Ser_619_Ala substitution) and 2CRHIS (bearing the sequence encoding His_6_ tag and an *Eco*RI site). (2) Using a mix of both the resulting PCR fragments as a template, and 2NFΔTM2 and 2CRHIS primers, an ORF fragment (containing a *Nsi*I site) was then obtained by high-fidelity overlap PCR. (3) An ES fragment encoding the expression and secretion system (P_Zn_*zitRexp4*_*SP*_) of pLB145 [4] was amplified using PznF and SPΔTM2R primers. Using a mix of ES and ORF fragments as a template and PznF and 2CRHIS primers, a high-fidelity overlap PCR was performed. Finally, using the resulting fragment as a template, ZitH2F and 2CRHIS primers and a A-tailing *Taq* polymerase (DNA Expand ROCHE), *htrA*_*2r*_ PCR fragment was obtained.

Each *htrA*_*1r*_ and *htrA*_*2r*_ PCR fragment was cloned into pGEM-T Easy Vector (Promega), according to the manufacturer’s instructions. After transformation of highly competent JM109 cells, selection on ampicillin, X-Gal and IPTG, and screening for white colonies, recombinant plasmids were extracted, insert size was checked by PCR, and the inserts were sequenced. The resulting plasmids were respectively named pVE8124 and pVE8125 (Table 1B). *htrA*_*1r*_ and *htrA*_*2r*_ fragments were respectively recovered from pVE8124 or pVE8125 plasmids by *Nsi*I + *Eco*RI or *Bam*HI + *Eco*RI double digestions. They were subcloned into pLB145 [4] digested by the same enzymes (Additional file 2: Figure S1). After transformation into strain MG1363, the resulting plasmids were checked by PCR and sequenced. The final plasmids were respectively named pVE8126 and pVE8127 (Table 1B).

### Recombinant protein production in *L. lactis*

Recombinant lactococcal cells were grown in 5 mL of M17 medium (buffered by 88 mM β-glycerophosphate) supplemented by 1% glucose, overnight in tubes. For growth in flasks, an overnight culture was diluted 100-fold in 280 mL of the same medium in a flask, and let to grow at 30°C. For growth in fermenters at controlled pH, serial tenfold dilutions of an overnight culture were grown overnight in M17 medium supplemented by 2% glucose), and cultures still in the exponential phase (OD_600_ between 0.4 and 0.7) were diluted 100-fold in 800 mL of preheated medium, either the same medium or a derivative, more strongly buffered, medium (176 mM β-glycerophosphate) to be grown in a fermenter (Biostat Q, Sartorius), at 30°C and at pH 6.5 (by addition of 5 N NaOH under shaking at 100 rpm).

When cultures reached an OD_600_ of 0.5 (or in one case, 2), induction was achieved by adding 500 µM EDTA, and cultures were further incubated for 4 h (or for 2 h in the case of the culture induced at OD_600_ 2). After centrifugation at low speed and at 4°C, supernatants were filtered on 0.22 µm, concentrated by about 25-fold by ultrafiltration (under a nitrogen pressure of less than 3 bars and at 4°C) using Millipore filters (Ultrafiltration Membranes NMWL 10,000), and stored at 4°C before purification.

### Protein purification

Dry resin (His-Select Nickel Affinity Gel, Sigma; 1 mL) was washed on a Econo-Pac column (Chromatography Columns; BIO-RAD) with 5 volumes of water and 10 volumes of buffer A (50 mM NaH_2_PO_4_, 300 mM NaCl, 10 mM Imidazole (Sigma^®^); pH 8.0), incubated for 15 min at 4°C, equilibrated with 10 volumes of buffer A and incubated for a further 15 min at 4°C. In parallel, each supernatant was diluted twofold in an equal volume of buffer B (100 mM NaH_2_PO_4_, 600 mM NaCl, 20 mM imidazole at pH 8.0). Resin (1 mL) and treated supernatant were mixed. After incubation overnight at 4°C, the mix was loaded onto the column and washed twice with buffer A. After addition of 3 mL of buffer C [50 mM NaH_2_PO_4_, 300 mM NaCl, 250 mM imidazole (Sigma^®^); pH 8.0] repeated three times, the three elution fractions were pooled, concentrated nine- to tenfold by ultrafiltration (to reach a final volume of 1 mL), dialysed against PBS buffer overnight at 4°C with shaking (Slide-A-Lyser^®^ Dialysis Cassette 0.5–3 mL/3,500 Da, Pierce^®^). Finally, glycerol was added to a final concentration of 10% before storage at −20°C. Protein concentration was determined by the Bradford method (Bio-Rad Protein Assay) according to the manufacturer’s instructions, using BSA (Protein Assay Standard II) as a standard. After each concentration or purification step, the protein fractions were analysed for protein purity and stability by Western blotting using antibodies against HtrA_1_ [23] or His_6_ (INVITROGEN).

### Protein analysis by mass spectrometry

rHtrA proteins were loaded on precasted 4–12% Bis–Tris Mini Gels (Invitrogen, France), SDS-PAGE runs were performed, and gels were stained with Coomassie blue (BioRad, Marnes-la-Coquette, France). Bands of interest were excised and protein identification was performed as previously described [24] using PAPPSO platform facilities (Jouy-en-Josas, France; http://pappso.inra.fr). Following SDS-PAGE migration and trypsinolysis, protein identification was performed querying MS/MS data against a in-house database containing rHtrA_1_ and rHtrA_2_ sequences (Additional file 3: Figure S2) together with the database for *L. lactis* subsp. *cremoris* strain MG1363 proteins (Uniprot, 2011/03/04; http://www.uniprot.org/uniprot/?query=organism:mg1363&fil=organism:%22Lactococcus%20lactis%20subsp.%20cremoris%20%28strain%20MG1363%29%20[416870]%22&sort=score) and a in-house contaminant protein database. The number of spectras attributed to each rHtrA protein was high, whereas no *L. lactis* protein could be detected.

### Secondary structure analysis by circular dichroism

Synchrotron radiation circular dichroism experiments, covering the UV spectral range from 190 to 305 nm, were carried out at the DISCO beam line of the Synchrotron SOLEIL (Saint Aubin, France; http://www.synchrotron-soleil.fr). Spectra were acquired at different temperatures, progressively increasing by 5°C in the 25–95°C range. CaF_2_ (Calcium Fluoride) 50 μm optical pathlength cells were loaded with 2 µL of each protein (rHtrA_1_ or rHtrA_2_) in a 50 mM Tris HCl (pH 7.5) and 50 mM NaCl buffer to reach a final protein concentration of 1 mg/mL. For each temperature curve, the mean of three spectra (acquired at 1 nm step per second between 190 and 305 nm) was calculated before subtraction of the baseline (by buffer subtraction) and set to zero between 255 and 260 nm. Mean spectra were calibrated to a standard solution of (+)-camphor-10-sulphonic acid (CSA), normalized and converted to Δε (molar circular dichroism, M^−1^ cm^−1^) using the software *CDtool* [60]. The thermal denaturation curve of each rHtrA protein was calculated at 207 nm, and the melting temperature (T_m_) was determined from a sigmoidal fit. Spectra were represented using Origin software (OriginLab, Northampton, MA, USA).

### Size exclusion chromatography coupled to multi angle light scattering

Purified rHtrA_1_ or rHtrA_2_ proteins (30 μL of samples at 1–4 mg/mL) were loaded on a KW-803 column (Shodex) equilibrated in PBS buffer at a 0.5 mL/min flow rate (Shimadzu HPLC system). Detection was performed using a MiniDAWN TREOS multi angle light scattering detector and an Optilab T-rEX differential refractometer (Wyatt Technology). Molar mass was calculated with the Astra 6.1.1.17 software, using a differential index of refraction (dn/dc) value of 0.183 mL/g.

### Protein folding assays

First, the refolding of two proteins, citrate synthase and α-glucosidase, was followed. Denaturation and renaturation reactions were carried out at 20°C. For both proteins, renaturation was initiated by pouring the renaturation solvent onto the unfolded protein under vortex agitation in Eppendorf polyethylene tubes. Citrate synthase was denatured at a concentration of 10 µM in 8 M urea, 50 mM Tris, 2 mM EDTA, 20 mM dithiothreitol pH 8.0 for 30 min. Renaturation was initiated by a 100-fold dilution in 40 mM Hepes, 50 mM KCl, 10 mM (NH_4_)_2_SO_4_, 2 mM potassium acetate, pH 8.0, in the absence of added protein or in the presence of DnaK, rHtrA_1_ or rHtrA_2_. The enzymatic activity of citrate synthase was measured as described previously [61]. α-Glucosidase was denatured at a concentration of 2 µM in 8 M urea, 0.1 M potassium phosphate, 1 mM EDTA, 20 mM dithiothreitol, pH 7.0 for 5–10 min. Renaturation was initiated by a 30-fold dilution in 40 mM Hepes–KOH, pH 7.8 at 20°C. The enzymatic activity of α-glucosidase was measured as described previously [61]. Concentrations of substrate proteins and chaperones were similar to those used by us [61] and others [62] for investing chaperone activities of GroEL and thioredoxin. Pig heart citrate synthase and *Saccharomyces cerevisiae* α-glucosidase were from Sigma. DnaK was purified as described in [61].

Second, the thermal aggregation of citrate synthase was followed. The native enzyme (80 µM) was diluted 100-fold in 40 mM Hepes, 50 mM KCl, 10 mM (NH_4_)_2_SO_4_, 2 mM potassium acetate, pH 8.0, at 44°C, in the absence of added proteins, or in the presence of DnaK, rHtrA_1_ or rHtrA_2_. Citrate synthase aggregation was monitored by measuring the absorbance at 650 nm as described in [63].

### Immunization of mice and Western blotting

OF1 mice were immunized with 10 μg of rHtrA_1_ or rHtrA_2_ co-injected with SP02 adjuvant (proprietary adjuvant of Sanofi Pasteur) by the subcutaneous route (0.2 mL) in the scapular girdle region at day 0, day 21 and day 36. Blood samples were collected under anaesthesia (see above) at day 0 and at day 61 at the retro-orbital sinus.

Staphylococcal culture pellets were lysed according to optical density. An equivalent of OD_680nm_ = 20 was lysed, for each pellet, in 250 µL of 2X lysis buffer: Tris–HCl 20 mM, Triton 1.2%, PMSF 1 mM, Halt Protease Inhibitor Single-Use Cocktail (Thermo Scientific) 1X, benzonase (Sigma) 5 U/µL and lysostaphin (Sigma) 100 µg/µL and incubated over night at 37°C. This suspension was heat inactivated 15 min at 95°C.

Samples were resolved by SDS 4–12%-PAGE (NuPAGE, Invitrogen) and transferred to nitrocellulose membranes (Transblot Transfert Medium, BioRad). Membranes were saturated with 5% skim milk in PBS buffer (2.7 mM KCl, 137 mM NaCl) overnight at 4°C. Filters were then incubated with 1:200 dilution of the primary antibody in 1% skim milk in PBS buffer for 1 h at room temperature, washed three times for 5 min with PBS with 0.05% Tween 20 (PBST). Filters were then incubated with 1:3,000 dilution of a Peroxidase AffiniPure F(ab’)2 fragment goat anti-mouse IgG (H + L) (Jackson ImmunoResearch Laboratories, USA) with 1% skim milk in PBS buffer for 1 h at room temperature and washed three times for 5 min as described above. Finally, the filters were washed 10 min in deionized water. Immunopositive bands were visualized using the Amplified Opti-4CN Kit (Bio-Rad Laboratories, Inc., USA) and were quantified using a densitometer (Genetools, Syngene, UK).

### Flow cytometry assay

The ability of polyclonal antisera elicited by the recombinant proteins to bind to the surface of live *S. aureus* strains was determined using flow cytometric detection of indirect fluorescence. Strain Lowenstein was grown at 37°C with shaking. Frozen bacteria were inoculated into 50 mL of appropriate medium and grown till exponential, late exponential or late stationary phases. A culture sample was centrifuged and washed once with PBS (Eurobio, Courtaboeuf, France). The final pellet was resuspended in PBS with 1% bovine serum albumin (BSA, Eurobio, Courtaboeuf, France) at a density of 10^8^ CFU/mL. 20 µL of dilutions of pooled serum were added to 20 µL of bacteria in 96 deep-well plates (Ritter, Schwabmunchen, Germany). For each serum, three dilutions were tested: 1/200, 1/2,000 and 1/20,000. The plate was incubated for 1 h at 37°C with shaking. The bacteria were centrifuged, washed once with PBS 1% BSA and resuspended with 100 µL of goat anti-mouse IgG F(ab’)2 conjugated to PE (Southern Biotech, Birmingham, USA) diluted 100-fold. The plate was incubated for 1 h at 37°C with shaking in the dark. The bacteria were washed twice with PBS 1% BSA. The fluorescent staining of bacteria was analyzed on a Cytomics FC500 flow cytometer (Beckman Coulter, Fullerton, USA). The fluorescent signal obtained for bacteria incubated with the specific polyclonal antisera was compared to the signal obtained for bacteria incubated with the corresponding negative control serum (buffer + SP02 adjuvant alone).

## Results and discussion

### Design of recombinant staphylococcal HtrA proteins

**Figure 1:**
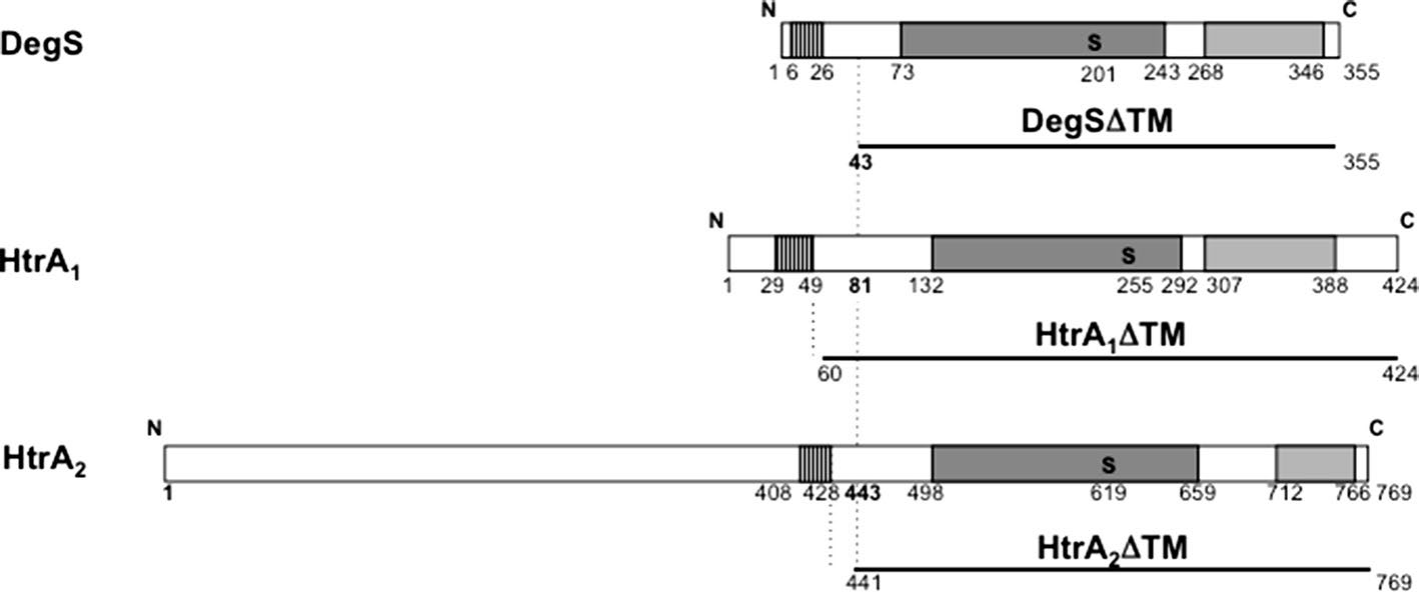
Architecture of staphylococcal HtrA proteins and design of soluble proteins. Three HtrA family members are shown: from *top* to *bottom*, *E. coli* DegS, *S. aureus* HtrA_1_ and HtrA_2_ proteins (strain COL). They display the typical domain organisation of the family: transmembrane, catalytic and PDZ domains are shown as hatched, *dark grey* and *light grey boxes* respectively, with their boundaries (residue position) indicated below. The catalytic Serine residue (S, in *bold*) is shown. The recombinant DegS form whose structure has been solved after N-terminal transmembrane domain deletion [64] is named DegSΔTM. A similar deletion strategy was applied to HtrA_1_ and HtrA_2_ proteins leading to N-terminally truncated proteins named HtrA_1_ΔTM and HtrA_2_ΔTM. All truncated protein forms are shown as *lines* with the position of their first and last residues in the corresponding WT sequence indicated.

In *S. aureus*, there are two transmembrane members of the HtrA family, HtrA_1_ and HtrA_2_ [23, 57] (for example in strain COL: Q5HF46 and Q5HH63, and data not shown for other published genomes; here, we provide the sequence of *htrA* genes from the clinical strain Lowenstein: Genbank BankIt1643789 htrA1_LOW KF322112 and BankIt1643789 htrA2_LOW KF322111) which are highly conserved between strains (more than 95 or 61% identity respectively). Even though HtrA_2_ bears a large N-terminal domain of unknown function [23], both HtrA_1_ and HtrA_2_ proteins display the typical family architecture [25, 26] (Figure 1) with three regions: from their N- to C-terminus, (1) a transmembrane domain as the export signal, (2) a catalytic domain with a characteristic His Asp Ser triad (His_144_, Asp_174_ and Ser_255_ in the case of HtrA_1_, and His_504_, Asp_534_ and Ser_619_ in the case of HtrA_2_) and (3) one PDZ domain (a protein–protein interaction domain named for the three proteins (PSD95, DLG1, and ZO-1) where it was initially discovered [26]). In both staphylococcal HtrA proteins, like in other family members of Gram-positive species, the C-terminal region encompassing the catalytic and PDZ domains is predicted to be extra-cellular (predicted C-out topology by HMMTOP, http://www.enzim.hu/hmmtop/html/submit.html).

For each HtrA protein of the staphylococcal strain COL, a soluble, proteolytically inactive and tagged form was produced (see Additional file 3: Figure S2). (1) The soluble, C-terminal region (devoid of the transmembrane domain, Figure 1) was fused in frame to a lactococcal signal peptide (see below, [4]) to be produced as a secreted protein. (2) The conserved catalytic Ser residue (Ser_255_ or Ser_619_ in the case of HtrA_1_ or HtrA_2_ respectively) was replaced by an Ala residue to abolish proteolytic activity, and thus to avoid the self-degradation previously observed for WT proteins when overproduced in a lactococcal *htrA* mutant strain [23]. (3) A His_6_ tag was fused at the C-terminus of each recombinant protein to facilitate its purification.

To delete the N-terminal transmembrane domain of each HtrA protein without affecting the overall protein folding, we exploited the strategy previously applied to produce a recombinant, soluble form of the *E. coli* trans-membrane protein DegS (sharing 31% identity with each of the staphylococcal HtrA proteins) whose structure could be solved [64]. The precise boundary of the deletion in the staphylococcal HtrA proteins was chosen to preserve, upstream of the region homologous to recombinant DegS (Figure 1), an N-terminal negatively charged residue to contribute to the negative charge at the N-terminus of the mature secreted rHtrA protein (see below).

### Lactococcal system for protein production and secretion

For protein production and secretion in *L. lactis*, we chose to clone the recombinant ORFs in pLB145, a lactococcal expression and secretion vector [4]. In the resulting plasmids (Additional file 2: Figure S1), recombinant ORFs are under the control of the lactococcal ZitR-regulated P_*zit*_ promoter [11, 19] so that their expression can be induced by the addition of EDTA [4, 11]. They are fused in frame to the coding sequence of the lactococcal signal peptide SP_Exp4_ (bearing two positively charged residues at its N-terminus) plus the two negatively charged residues present at its C-terminus in the endogenous mature Exp4 protein [4, 20]. This fusion strategy preserves both the natural charges around SP_Exp4_ to ensure its correct insertion into the membrane (according to the positive-inside rule [65, 66]), and its natural cleavage site for the lactococcal signal peptide peptidase. Finally, this fusion strategy should ensure efficient secretion both at the translocation and release steps.

### Production and secretion of recombinant proteins in *L. lactis*, and purification

**Figure 2:**
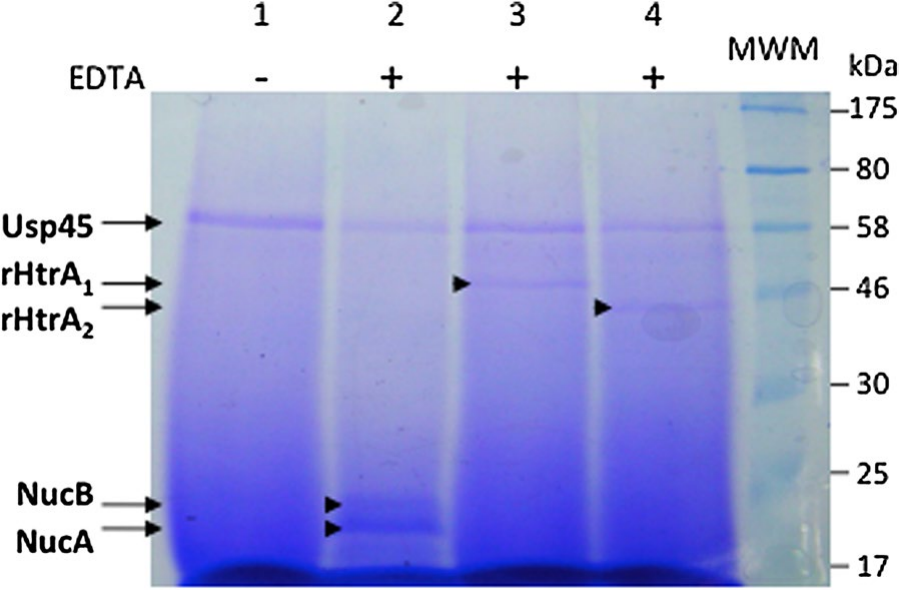
Production of secreted rHtrA proteins in *L. lactis.* rHtrA_1_ and rHtrA_2_ proteins, together with a recombinant form of the staphylococcal nuclease [4] as a positive control, were produced and secreted in *L. lactis*. Recombinant strains [MG1363(pLB145) in *lanes 1* and *2*, MG1363(pVE8126) in *lane 3* and MG1363 (pVE8127) in *lane 4*] were grown to the exponential phase in rich M17 medium supplemented with glucose and buffered with β-glycerophosphate, in flasks. EDTA at 500 μM was added to the cultures to induce (*lanes* *2*–*4*) or not (*lane 1*, as a negative control) recombinant protein production. After further growth, culture supernatants were recovered, concentrated, precipitated and finally subjected to SDS-PAGE and Coomassie Brilliant Blue staining. Culture supernatants all show the major lactococcal secreted protein, Usp45 [9] either alone (*lane 1*) or together with one of the recombinant proteins (*lanes 2*–*4*). Usp45 (*lanes 1*–*4*), rHtrA_1_ (*lane 3*), rHtrA_2_ (*lane 4*) and for recombinant staphylococcal nuclease (*lane 2*), both the secreted form, NucB, and its maturation product (released by lactococcal HtrA protease), NucA [4, 10], are indicated by *arrows.*

**Figure 3:**
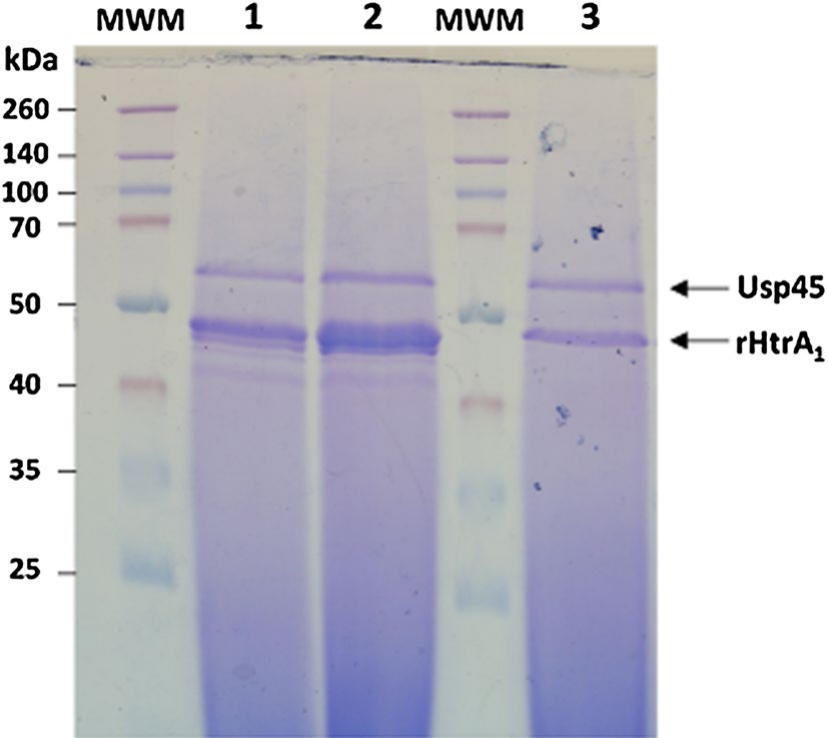
Optimization of rHtrA_1_ protein production in *L. lactis.* Growth conditions were optimized in order to improve protein yield. Strain MG1363(pVE8126) was grown in M17 medium supplemented with 2% glucose and buffered with β-glycerophosphate at two different concentrations (88 mM in *lanes 1* and *3*, and 176 mM in *lane 2*). Growth was performed either in fermenters at controlled pH (in 800 mL of medium, *lanes 1* and *2*) or in flasks (in 280 mL of medium, *lane 3*). Exponential phase cultures were induced by addition of EDTA at 500 μM, culture supernatants were concentrated and, after quantification, proteins were subjected to SDS-PAGE and stained by Coomassie Brilliant Blue. *MWM* molecular weight marker.

**Figure 4:**
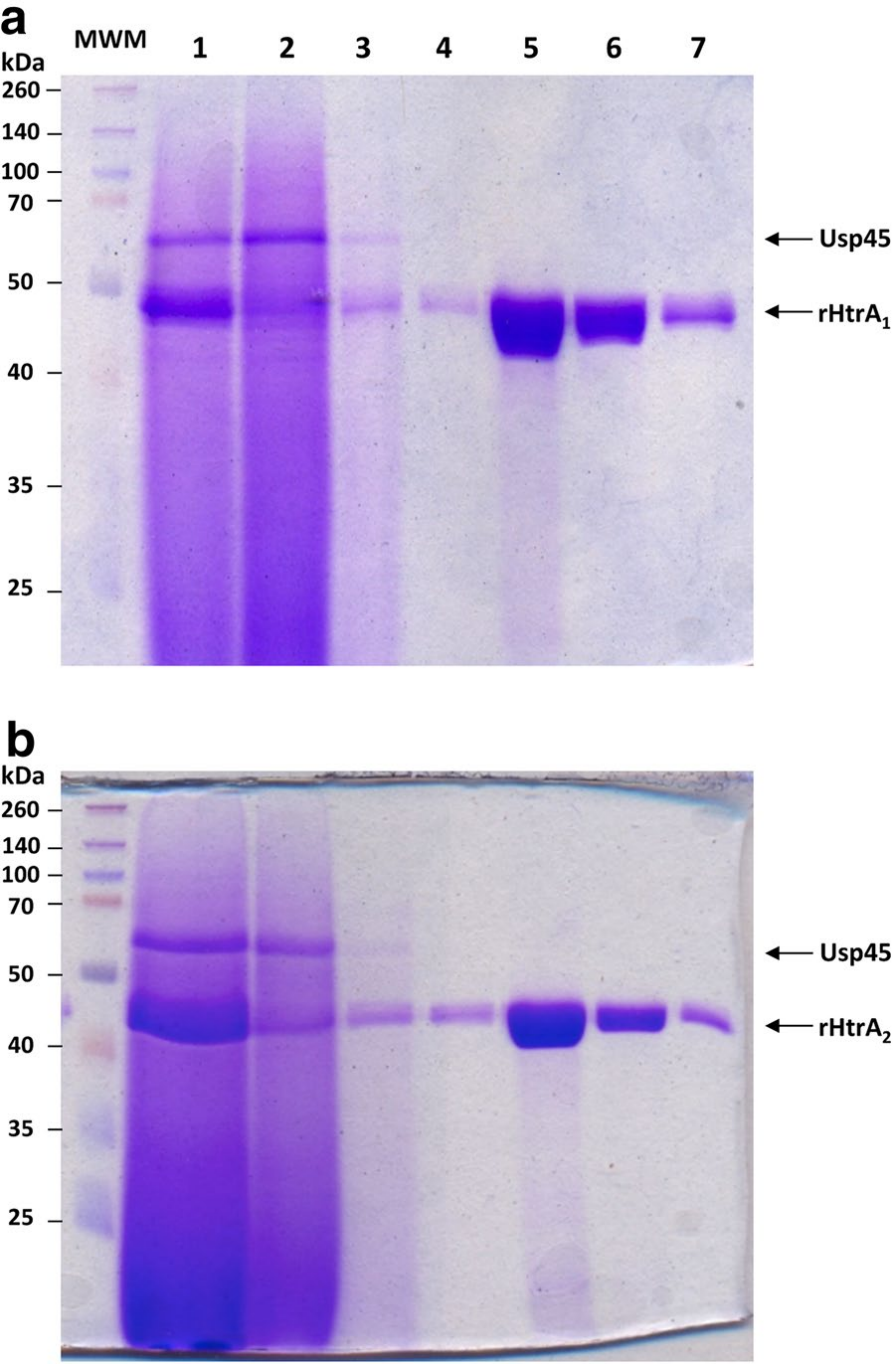
Purification of rHtrA proteins. Each rHtrA protein (rHtrA_1_ in **a** and rHtrA_2_ in **b**), after production and secretion in *L. lactis* (by either strain MG1363(pVE8126) in **a** or strain MG1363(pVE8127) in **b**) grown in fermenter as described in Figure 3, was purified by affinity. At each purification step, an SDS-PAGE analysis followed by Coomassie Brilliant Blue staining was performed. *Lane 1* concentrated supernatant from an induced culture after growth in fermenter, *Lane 2* flow through, *Lanes 3* and *4* washing number 1 and 2, *Lanes* *5*–*7* elution fractions number 1–3. *MWM* molecular weight marker.

The efficiency of recombinant protein production and secretion in an *L. lactis* WT strain (strain MG1363) was followed. After growth of the recombinant strains (bearing the recombinant pLB145-derived plasmids pVE8126 and pVE8127) in a rich medium up to the exponential growth phase and induction by EDTA addition, the culture supernatants were found to contain, in similar amounts as the major lactococcal secreted protein, Usp45 [9], an additional protein of the expected size (Figure 2), which could specifically be recognized by anti-HtrA_1_ [23] or anti-His_6_ antibodies (data not shown). Each secreted recombinant rHtrA protein was found to be stable in the culture supernatant of the otherwise WT host strain (Figures 2, 3 and 4a, lane 1 and Figure 4b, lane 1). This result is interesting in two ways. First, as the proteolytic activity and self-degradation ability of staphylococcal WT HtrA proteins are supported by several lines of evidence [23, 57], the stability of substituted rHtrA(Ser-Ala) proteins strongly suggests that the mutation of the main catalytic residue leads to protease inactivation, as expected. Second, as many heterologous and/or recombinant proteins are degraded by the endogenous HtrA protease in a lactococcal WT background [4, 10, 22], here, the resistance of rHtrA proteins to this endogenous protease strongly suggests that their translocation across the cytoplasmic membrane is efficient and quick, without accumulation of unfolded and degradation-prone intermediates. Our carefully designed protein fusion strategy (see above) therefore proved to create secretion-prone precursors that were translocated and released without being degraded. Carefully designed secretion in *L. lactis* can provide stable proteins and preclude the use of an *htrA* mutant devoid of surface proteolytic activity as the host strain although in some cases, probably depending on the induction level and/or on the intrinsic folding ability of the protein, this use can turn out to be necessary (for example the *Staphylococcus hyicus* WT lipase, a naturally secreted protein, is extensively degraded in *L. lactis* except in an *htrA* mutant [4]).

Growth conditions were then optimized to increase biomass and thus protein yield (Figure 3). Lactococcal recombinant strains were grown in fermenters at controlled pH. The rich medium used for lactococcal culture was optimized by increasing the concentration of glucose and β-glycerophosphate buffer in order to prevent bacterial lysis due to glucose starvation (data not shown, and Pascal Loubière, INRA Toulouse, personal communication) and to decrease the need for NaOH addition to control the pH, respectively. After EDTA induction in exponentially growing cultures, the recombinant proteins were secreted in higher amounts when recombinant lactococcal cells were grown in fermenters rather than in flasks [for rHtrA_1_, compare lane 3 and lane 1 in Figure 3, and for both rHtrA_1_ and rHtrA_2_ proteins, compare their relative amounts using Usp45 protein as a standard between Figures 2 and 4: in contrast to the situation after growth in flasks (Figure 2 lane 3 for rHtrA_1_ and lane 4 for rHtrA_2_), each rHtrA protein became the major secreted protein in fermenters (Figure 4a, lane 1 for rHtrA_1_ and Figure 4b, lane 1 for rHtrA_2_)]. In fermenters, a highly buffered medium further improved secretion efficiency (for rHtrA_1_, compare lane 1 and lane 2 in Figure 2, and data not shown for rHtrA_2_). Finally, for rHtrA protein production, recombinant lactococcal strains were grown in small-scale fermenters and rHtrA proteins were purified by chromatography affinity from the culture supernatants (Figure 4), concentrated, dialyzed and quantified by Bradford analysis. Using the optimized medium, protein yields were 2.5 and 2.2 mg/L for rHtrA_1_ and rHtrA_2_ respectively, and in total, about 7 mg of each protein could be obtained. The identity of purified rHtrA proteins was confirmed by trypsinolysis followed by mass spectrometry (Table 2). *L.* *lactis* thus proved to be an efficient cell factory to produce the soluble C-terminal region of trans-membrane HtrA proteins as secreted and stable forms (even after conservation at −20°C; data not shown).

**Table 2:** rHtrA protein identity

Description	Coverage (%)	Spectra	Unique peptides	log(E value)
rHtrA_1_	64	129	30	−157
rHtrA_2_	80	235	45	−298

Purified rHtrA proteins were submitted to trypsinolysis and mass spectrometry, and the results are shown.

### rHtrA proteins are correctly folded

**Figure 5:**
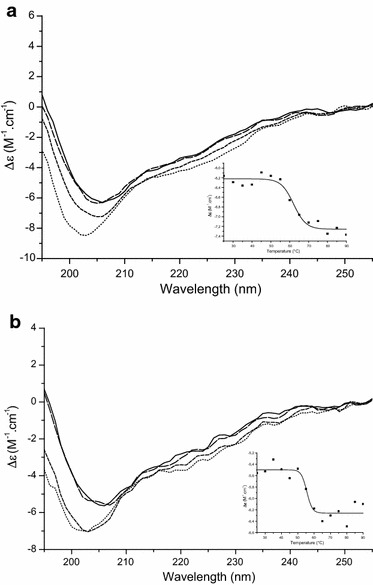
Characterization of rHtrA proteins by circular dichroism. SRCD spectra of rHtrA_1_ (**a**) and rHtrA_2_ (**b**) are shown at various temperatures: 25°C (*solid line*), 45°C (*large dashed line*), 65°C (*short dashed line*), and 85°C (*dotted line*). In each case, Δε (molar circular dichroism) at 207 nm was plotted against temperature and this graph is shown as an *inset*.

In order to characterize the secondary structure of purified rHtrA proteins, they were studied by synchrotron radiation circular dichroism (SRCD) spectroscopy at different temperatures (Figure 5). CD profiles of both proteins are similar to each other, and the protein secondary structures are probably mainly random coil (see minima around 202–205 nm in Figure 5) and some beta sheet (see shoulder around 215 nm in Figure 5), as expected for HtrA family members [67] and as previously described for another recombinant HtrA protein [68]. Staphylococcal rHtrA proteins share similar CD profiles with other recombinant HtrA proteins, in particular with an N-terminally truncated and inactive form of the periplasmic HtrA protein from *Haemophilus influenzae* [68], and, to a smaller extent, with a truncated, soluble form of the mitochondrial transmembrane HtrA2-Omi protein (after transmembrane domain deletion) [69]. The secondary structure profile of rHtrA proteins did not display any significant change between 25 and 45°C, indicating that they are folded under these conditions. Thermal denaturation and loss of secondary structure was observed above 60°C for rHtrA_1_ [melting temperature (Tm): 62.1°C ± 1.3 Figure 5a inset] and above 55°C for rHtrA_2_ (Tm: 55.5°C ± 1.4 Figure 5b inset). Similar results were previously obtained for recombinant HtrA proteins from *H. influenzae* [68] and *E. coli* [70].

**Figure 6:**
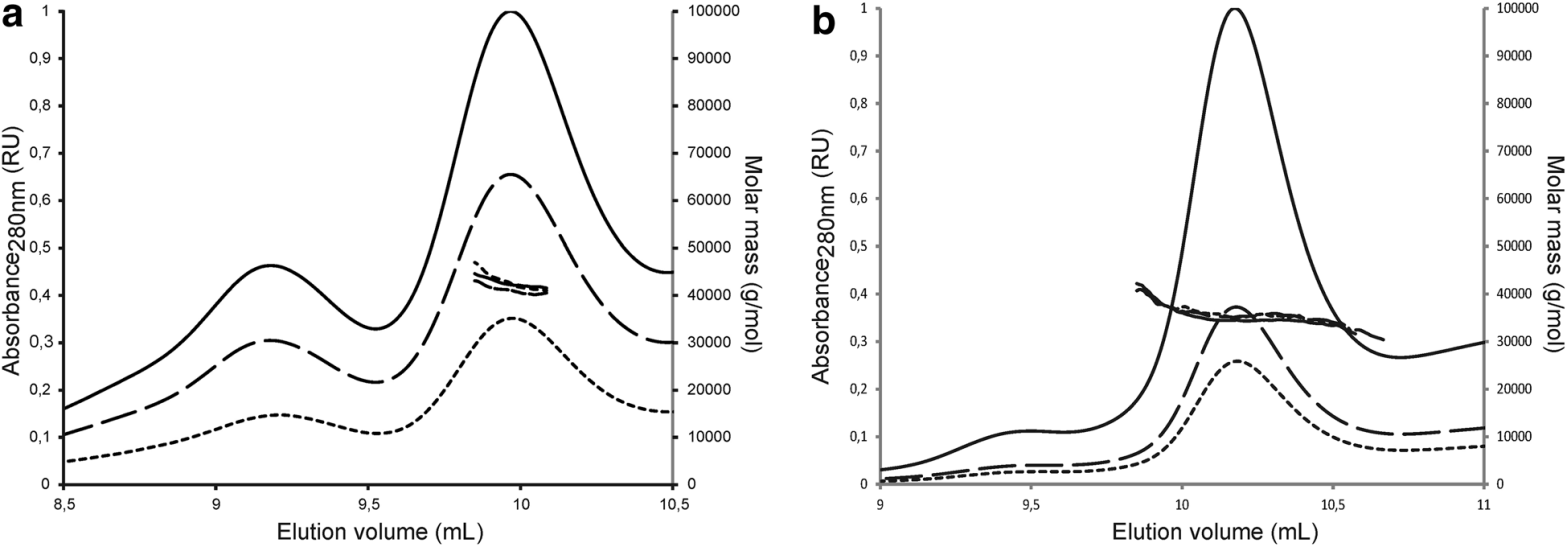
SEC-MALS analysis of purified rHtrA proteins. SEC-MALS analysis of (**a**) purified rHtrA_1_ (40 kDa) and (**b**) rHtrA_2_ (39 kDa), proteins is shown. rHtrA proteins at 1 mg/mL (*small dash line*), 2 mg/mL (*large dash line*) and 4 mg/mL (*solid line*) were loaded on a 15 mL KW-803 column (Shodex). Absorbance at 280 nm (on the *left*) and molar mass (on the *right*) are plotted as a function of the elution volume. Results obtained with rHtrA_1_ were confirmed using a Superdex 200 increase 10/300 GL column (GE, data not shown). *RU* relative unit.

Protein oligomeric status was then studied by size exclusion chromatography coupled to multi angle light scattering (SEC-MALS, at three different protein concentrations, Figure 6). They were found to be mainly monomeric in solution, in contrast to all other family members, known to be organized at least as homotrimers, both in solution and as crystals (see [64] for an example) or even, for some of them, as large size multimers of homotrimers [26]. Further studies will be needed to establish whether staphylococcal proteins are unable to trimerize.

### rHtrA proteins are active chaperones

**Figure 7:**
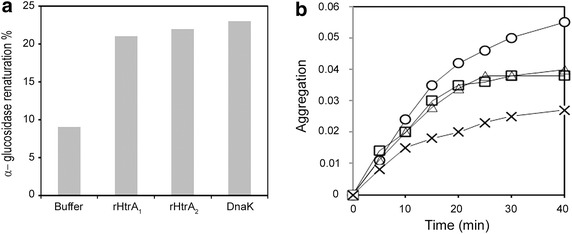
Chaperone properties of rHtrA proteins. **a** Refolding of urea-denatured α-glucosidase in the presence of rHtrA_1_ or rHtrA_2_. α-Glucosidase was denatured in urea and then renatured for 20 min by dilution of the denaturant as described under “Methods”, at a concentration of 0.07 µM in the absence of additional protein and in the presence of either 5 µM rHtrA_1_, 5 µM rHtrA_2_ or 5 µM DnaK. **b** Thermal aggregation of citrate synthase in the presence of rHtrA_1_ or rHtrA_2_. The kinetics of citrate synthase aggregation was determined by light scattering at 650 nm. Native citrate synthase was diluted to a final concentration of 0.8 µM at 44°C, as described under “Methods”, in the absence of additional protein (*circles*), or in the presence of 5 µM rHtrA_1_ (*triangles*), 5 µM rHtrA_2_ (*squares*) or 5 µM DnaK (*crosses*).

The chaperone activity of both recombinant proteins was then tested as many HtrA proteins are dual proteins displaying both proteolytic and folding activities [25, 27], and as their chaperone activity can be demonstrated when their catalytic Serine residue is substituted to an Alanine [71]. We first examined the renaturation of urea-unfolded α-glucosidase in the presence of purified rHtrA_1_ and rHtrA_2_. Maximal recovery of α-glucosidase activity was 9% in the absence of chaperone, 21% in the presence of 5 µM rHtrA_1_, 22% in the presence of 5 µM rHtrA_2_, and 23% in the presence of 5 µM DnaK (Figure 7a) as a positive control [61]. We also investigated the renaturation of urea-unfolded citrate synthase in the presence of rHtrA_1_ and rHtrA_2_. The maximal recovery of citrate synthase activity was 8% in the absence of chaperone, 12% in the presence of 5 µM rHtrA_1_, 13% in the presence of 5 µM rHtrA_2_ and 18% in the presence of 5 µM DnaK (not shown). On the contrary, as previously reported [72], ovalbumin and lysozyme were unable to stimulate the renaturation of either citrate synthase or α-glucosidase (not shown). Thus, both rHtrA_1_ and rHtrA_2_ increased the productive folding of urea-denatured α-glucosidase and citrate synthase.

We then investigated the function of rHtrA_1_ and rHtrA_2_ under heat shock conditions. As reported previously [63, 72], citrate synthase loses its native conformation and undergoes aggregation during incubation at 44°C. As shown in Figure 7b, 5 µM rHtrA_1_ or 5 µM rHtrA_2_ reduced citrate synthase aggregation by 30%, whereas 5 µM DnaK reduced citrate synthase aggregation by 49%. On the contrary, ovalbumin and lysozyme, as previously reported [72], were inefficient in protecting citrate synthase from thermal denaturation (not shown). These results suggest that rHtrA_1_ and rHtrA_2_ can interact with partially unfolded proteins and protect them against thermal denaturation.

Together, these results demonstrate for the first time that the soluble domains of staphylococcal HtrA proteins possess chaperone activity in vitro. This activity is in the case of HtrA_2_ protein independent of the long, N-terminal domain of unknown function [23], at least in vitro, and further studies will be needed to determine if this domain could contribute to envelope protein folding in *S. aureus* and/or to virulence in vivo. Finally, our results indicate that rHtrA proteins are correctly folded, and thus strongly suggest that they should expose conformational epitopes relevant for vaccine applications.

### Purified rHtrA proteins are immunogenic in mice and WT HtrA_1_ protein is exposed at the staphylococcal cell surface

**Figure 8:**
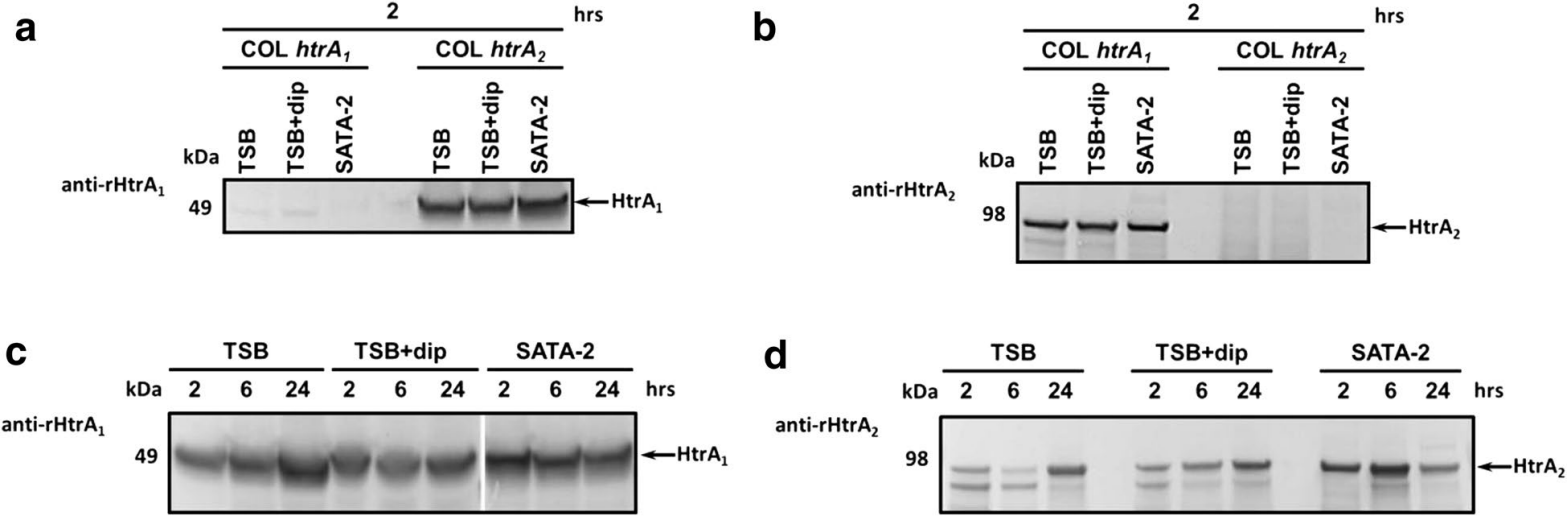
Expression of WT HtrA proteins in *S. aureus.* Immunoblot analysis using polyclonal anti-rHtrA_1_ (**a**, **b**) or anti-rHtrA_2_ (**c**, **d**) sera was performed on cell lysates of different *S. aureus* strains: each *htrA* mutants of strain COL (designated as COL *htrA*_*1*_ and COL *htrA*_*2*_ [57], **a**, **b**) and WT strain Lowenstein (**c**, **d**). All strains were grown in two media (TSB, SATA-2) and in the first line, under two different conditions (TSB, TSB + dipyridyl corresponding to the addition of chelator), till different growth phases: the exponential phase (2 h) for *htrA* mutants of strain COL (**a**, **b**) and, for WT strain Lowenstein (**c**, **d**), the exponential (2 h), early stationary (6 h) and late stationary (24 h) phases. HtrA_1_ was found to be completely stable (**a**, **c**), whereas HtrA_2_ protein underwent a limited proteolysis giving rise to one minor degradation product of high molecular weight in the cells (**b**, **d**), as confirmed by the absence of proteolytic products in culture supernatants. *S. aureus* HtrA_1_ and HtrA_2_ thus behave differently from *Bacillus subtilis* HtrA (YkdA) protein (YkdA) which is known to be released into the supernatant as a processed form, devoid of its transmembrane domain [73].

WT HtrA proteins were then characterized to get insights on their expression and localization in staphylococcal cells. After injection of folded rHtrA_1_ or rHtrA_2_ protein in mice in the presence of an adjuvant (SP02, a proprietary adjuvant of Sanofi Pasteur), sera with high (>5 Log) IgG1 and IgG2a titers could be obtained, indicating that rHtrA proteins are immunogenic. The specificity of the raised polyclonal antibodies was demonstrated by Western blot analysis of cellular extracts from staphylococcal *htrA* mutant strains grown to the exponential phase (Figure 8a, b).

These antibodies were then used to study the expression and cell surface localization of HtrA proteins in *S. aureus* after growth under different conditions. In a preliminary experiment, expression was studied in two strains (strain COL and strain Lowenstein which is responsible for systemic infections) grown in two media: a defined medium (containing a high salt concentration), and a complex standard medium supplemented or not with 2.2′ Dipyridyl (a chelator leading to iron depletion) as high salt concentration and iron depletion are close to the conditions faced by *S. aureus* in the host. Western blotting allowed detecting full-length HtrA_1_ and HtrA_2_ proteins in the cells in similar amounts under all conditions (Figure 8), indicating a constitutive expression under the tested conditions and the absence of extensive degradation, as confirmed by the analysis of culture supernatants (data not shown).

**Figure 9:**
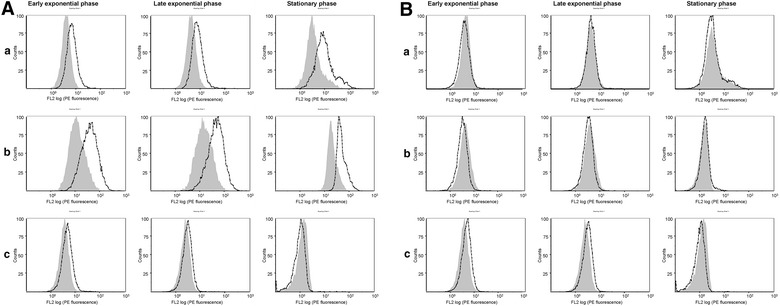
Cytometry analysis of WT HtrA protein exposure at the staphylococcal cell surface. The cell surface exposure of WT HtrA_1_ (**A**) and HtrA_2_ (**B**) proteins from strain Lowenstein was analyzed by flow cytometry. Staphylococcal cells were grown either in TSB medium (*a*), TSB supplemented with 2,2′ Dipyridyl 1 mM (*b*), or SATA-2 medium (*c*) till early exponential phase, late exponential phase or late stationary phase. *Shaded* and *white areas* represent cells incubated with antibodies against SP02 adjuvant or each rHtrA protein (rHtrA_1_ in **A** and rHtrA_2_ in **B**) respectively.

Finally, the exposure of the HtrA protein C-terminal region at the staphylococcal cell surface in strain Lowenstein was tested by cytometry analysis using the mice polyclonal antibodies. In staphylococcal cells grown in the complex medium (supplemented or not with 2.2′ Dipyridyl), HtrA_1_ protein could be detected at the cell surface (Figure 9A) confirming its predicted topology and the cell surface exposure of its C-terminal region, in agreement with our previous demonstration of its extra-cellular proteolytic activity [23]. On the contrary, HtrA_2_, although produced under the same growth conditions (Figure 8D), was not accessible to antibodies added from the medium (Figure 9B), suggesting that the HtrA_2_ C-terminal region might remain embedded in the staphylococcal cell wall. These results suggest that, in the perspective of vaccine development, rHtrA_1_ protein might be a better candidate than rHtrA_2_ protein, even though further studies will be needed to study their expression and antigenicity in vivo in the host.

## Conclusions

In this study, the C-terminal region of staphylococcal HtrA transmembrane proteins could efficiently be produced and secreted in *L. lactis* as correctly folded and stable forms that were easily purified from the culture medium in one step. *L. lactis* was demonstrated to be an efficient cell factory with respect to protein quality, in terms of both purity and folding, in particular in the case of a surface-exposed antigen, like staphylococal HtrA_1_ protein. Our results indicate that to produce proteins of high quality and purity for medical applications, *L. lactis* is an efficient and competitive alternative to *E. coli* and *B. subtilis* hosts.

## Additional files

Additional file 1:
**Table S1.** Primers used in this study Additional file 2:
**Figure S1.** Plasmids used for the production and secretion of rHtrA proteins. Plasmids pVE8126 (on the left) and pVE8127 (on the right) for the production and secretion rHtrA proteins were constructed by cloning recombinant *htrA*_*1r*_ (on the left) and *htrA*_*2r*_ (on the right) ORFs into pLB145: *htrA*_*1r*_ was cloned in place of *nuc* by *Nsi*I and *Eco*RI double digestion, whereas *htrA*_*2r*_ was cloned in place of *exp4*_*SP*_ *-nuc* by *Bam*HI and *Eco*RI double digestion. The proteins encoded by each *htrA*_*r*_ ORF are represented down below. For both of them, the catalytic and PDZ domains, together with the His_6_-tag are shown as dark grey, light grey and black boxes respectively, with their boundaries indicated, and the Alanine (A) substituting the catalytic residue is also indicated in bold with its position. Whereas *htrA*_*2r*_ ORF encodes the entire protein precursor with SP_Exp4_ signal peptide (horizontally hatched box), *htrA*_*1r*_ is fused in frame to *exp4*_*SP*_ (encoding SP_Exp4_) by cloning, leading to the *exp4*_*SP*_ *-htrA*_*1r*_ fusion encoding the protein precursor. Additional file 3:
**Figure S2.** rHtrA protein sequence. rHtrA proteins (rHtrA1 in panel A and rHtrA2 in panel B) are the mature secreted forms of hybrid precursors after the clivage of lactococcal SPExp4 signal-peptide (MKKINLALLTLATLMGVSST AVVFA) [20]. Both rHtrA proteins retain at their N-terminus, the first two negatively charged residues of mature secreted Exp4 form (in blue). At their C-terminus, they both bear a His6 tag (in brown). In both of them, the catalytic residue (Serine 255 in HtrA_1_ and Serine 619 in HtrA_2_) has been substituted to an Alanine residue (in red). N-terminally truncated HtrA proteins (after transmembrane deletion) are shown in black: in A, HtrA_1_ΔTM is HtrA_1_ (Q5HF46) starting at Aspartate 60 and in B, HtrA_2_ΔTM is HtrA_2_ (Q5HH63) starting at Aspartate 441 (see Figure 1).
