# Supplementary material for: Production, secretion and purification of a correctly folded staphylococcal antigen in Lactococcus lactis
Source: Microb Cell Fact. 2015 Jul 16;14:104. doi: 10.1186/s12934-015-0271-z (PMC4502909; doi:10.1186/s12934-015-0271-z)
Supplement: Additional file 1: — Table S1. Primers used in this study [file 12934_2015_271_MOESM1_ESM.docx]

| Name | Orientation | Sequence |
| --- | --- | --- |
| 1NFΔTM2 | Forward | CCA ATG CAT CA GAT GGT TCA ACT GTT CAG AC |
| 1IFS>A | Forward | GCT ATT AAC CCA GGT AAC GCA GGT GGC GCA TTA GTA G |
|  |  |  |
| 1IRS>A | Reverse | ATC TAC TAA TGC GCC ACC TGC GTT ACC TGG GTT AAT AGC |
|  |  |  |
| 1CRHIS | Reverse | CCG GAA TTC **TTA GTG ATG GTG ATG ATG ATG** TCT AAA GAA ATC TCT ATC |
|  |  |  |
| 2NFΔTM2 | Forward | CC GTT GTA TTT GCT GAT GAT GAT GCA GAT GCT CAA AAA TAT AC |
|  |  |  |
| 2IFS>A | Forward | CA TCA GTA AAT CCA GGT AAC GCG GGT GGT GCT GTC GTC AAT AG |
|  |  |  |
| 2IRS>A | Reverse | ATT GAC GAC AGC ACC ACC CGC GTT ACC TGG ATT TAC TGA TG |
|  |  |  |
| 2CRHIS | Reverse | CCG GAA TTC **TTA GTG ATG GTG ATG ATG ATG** TTT TAG TTT AAT ATT AAT TTC |
|  |  |  |
| PznF | Forward | GCT CCA AGA TCT TTG ATC AAG |
|  |  |  |
| SPΔTM2R | Reverse | GT ATA TTT TTG AGC ATC TGC ATC ATC ATC AGC AAA TAC AAC GG |
|  |  |  |
| ZitH2-F | Forward | TCG ATG AAG GAT CCG GTA CC |
|  |  |  |
| pLB145F | Forward | TTA TTG TTT TGA TGT TCG GC |
|  |  |  |
| pLB145R | Reverse | CGA CGG TAT CGA TAA GCT TG |
|  |  |  |
