# Supplementary figures and images for: Production, secretion and purification of a correctly folded staphylococcal antigen in Lactococcus lactis
Source: Microb Cell Fact. 2015 Jul 16;14:104. doi: 10.1186/s12934-015-0271-z (PMC4502909; doi:10.1186/s12934-015-0271-z)

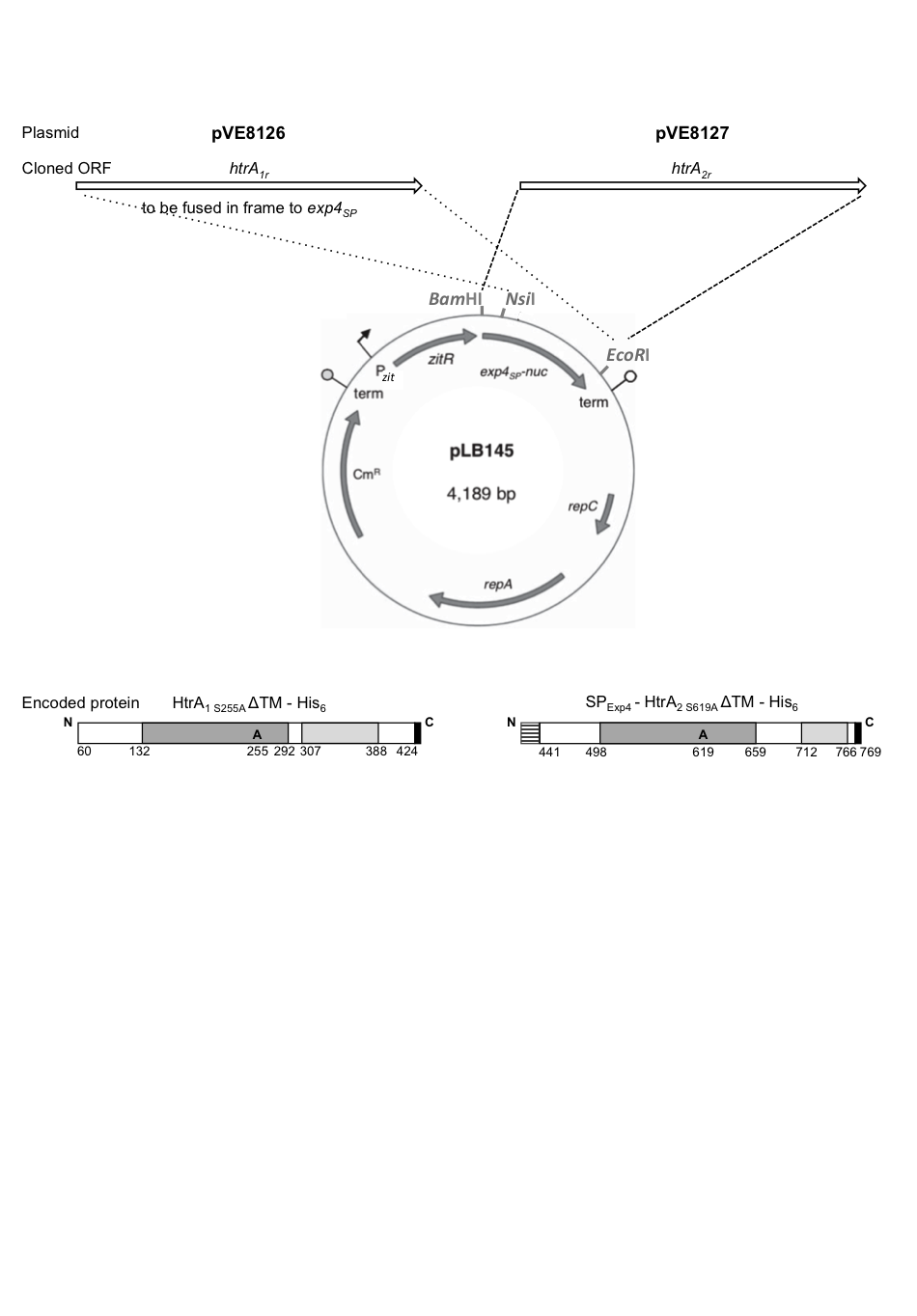

Supplement: Additional file 2: — Figure S1. Plasmids used for the production and secretion of rHtrA proteins. Plasmids pVE8126 (on the left) and pVE8127 (on the right) for the production and secretion rHtrA proteins were constructed by cloning recombinant htrA 1r (on the left) and htrA 2r (on the right) ORFs into pLB145: htrA 1r was cloned in place of nuc by NsiI and EcoRI double digestion, whereas htrA 2r was cloned in place of exp4 SP -nuc by BamHI and EcoRI double digestion. The proteins encoded by each htrA r ORF are represented down below. For both of them, the catalytic and PDZ domains, together with the His6-tag are shown as dark grey, light grey and black boxes respectively, with their boundaries indicated, and the Alanine (A) substituting the catalytic residue is also indicated in bold with its position. Whereas htrA 2r ORF encodes the entire protein precursor with SPExp4 signal peptide (horizontally hatched box), htrA 1r is fused in frame to exp4 SP (encoding SPExp4) by cloning, leading to the exp4 SP -htrA 1r fusion encoding the protein precursor. [file 12934_2015_271_MOESM2_ESM.tiff]
