# Supplementary material for: Production, secretion and purification of a correctly folded staphylococcal antigen in Lactococcus lactis
Source: Microb Cell Fact. 2015 Jul 16;14:104. doi: 10.1186/s12934-015-0271-z (PMC4502909; doi:10.1186/s12934-015-0271-z)
Supplement: Additional file 3: — Figure S2. rHtrA protein sequence. rHtrA proteins (rHtrA1 in panel A and rHtrA2 in panel B) are the mature secreted forms of hybrid precursors after the clivage of lactococcal SPExp4 signal-peptide (MKKINLALLTLATLMGVSST AVVFA) [20]. Both rHtrA proteins retain at their N-terminus, the first two negatively charged residues of mature secreted Exp4 form (in blue). At their C-terminus, they both bear a His6 tag (in brown). In both of them, the catalytic residue (Serine 255 in HtrA1 and Serine 619 in HtrA2) has been substituted to an Alanine residue (in red). N-terminally truncated HtrA proteins (after transmembrane deletion) are shown in black: in A, HtrA1ΔTM is HtrA1 (Q5HF46) starting at Aspartate 60 and in B, HtrA2ΔTM is HtrA2 (Q5HH63) starting at Aspartate 441 (see Figure 1). [file 12934_2015_271_MOESM3_ESM.docx]

**A. rHtrA_1_ protein**

**D D** **A S** D_60_ G S T V Q T T N N K G G N Q L D G Q S K K F G T V H E M I K S V S P T I V G V I N M Q K A S S V D D L L K G K S S K P S E A G V G S G V I Y Q I N N N S A Y I V T N N H V I D G A N E I R V Q L H N K K Q V K A K L V G K D A V T D I A V L K I E N T K G I K A I Q F A N S S K V Q T G D S V F A M G N P L G L Q F A N S V T S G I I S A S E R T I D A E T T G G N T K V S V L Q T D A A I N P G N **A_255_** G G A L V D I N G N L V G I N S M K I A A T Q V E G I G F A I P S N E V K V T I E Q L V K H G K I D R P S I G I G L I N L K D I P E E E R E Q L H T D R E D G I Y V A K A D S D I D L K K G D I I T E I D G K K I K D D V D L R S Y L Y E N K K P G E S V T V T V I R D G K T K E V K V K L K Q Q K E Q P K R Q S R S E R Q S P G Q G D R D F F R **H H H H H H**

**B. rHtrA_2_ protein**

**D D** D_441_ A D A Q K Y T T T M K N A N N T V K S V V T V E N E T S K D S S L P K D K A S Q D E V G S G V V Y K K S G D T L Y I V T N A H V V G D K E N Q K I T F S N N K S V V G K V L G K D K W S D L A V V K A T S S D S S V K E I A I G D S N N L V L G E P I L V V G N P L G V D F K G T V T E G I I S G L N R N V P I D F D K D N K Y D M L M K A F Q I D A S V N P G N **A_619_** G G A V V N R E G K L I G V V A A K I S M P N V E N M S F A I P V N E V Q K I V K D L E T K G K I D Y P D V G V K M K N I A S L N S F E R Q A V K L P G K V K N G V V V D Q V D N N G L A D Q S G L K K G D V I T E L D G K L L E D D L R F R Q I I F S H K D D L K S I T A K I Y R D G K E K E I N I K L K **H H H H H H**
